# Creation of a rectal cancer registry in Italy by the Advanced International Mini-Invasive Surgery (AIMS) academy clinical research network

**DOI:** 10.12688/f1000research.20702.1

**Published:** 2019-10-10

**Authors:** Giulio M. Mari, Pietro Achilli, Dario Maggioni, Jacopo Crippa, Andrea T.M. Costanzi, Mauro A. Scotti, Vittorio Giardini, Mattia Garancini, Eugenio Cocozza, Giacomo Borroni, Ilaria Benzoni, Mario Martinotti, Luigi Totaro, Matteo Origi, Michele Mazzola, Giovanni Ferrari, Antonio Ziccarelli, Roberto Petri, Vincenzo Bagnardi, Giacomo Pugliese, Antonello Forgione, Raffaele Pugliese, AIMS Academy Clinical Research Network

**Affiliations:** 1 General Surgery Department, Desio Hospital, Desio, Italy; 2 General Surgery Residency Program, University of Milan, Milan, Italy; 3 General Surgery Department, San Gerardo Hospital, Monza, Italy; 4 General Surgery Department, Varese Hospital, Varese, Italy; 5 Department of Surgery, Cremona Hospital, Cremona, Italy; 6 General Surgery Department, Niguarda Hospital, Milan, Italy; 7 General Surgery Department, AOU "SSMM della Misericordia", Udine, Italy; 8 Department of Statistics and Quantitative Methods, University of Milan-Bicocca, Milan, Italy; 9 AIMS Academy Clinical Research Network, Advanced International Mini-Invasive Surgery (AIMS) Academy, Milan, Italy

**Keywords:** Rectal surgery, registry, network

## Abstract

**Background: **The management of rectal cancer is multimodal and involves a multidisciplinary team of cancer specialists with expertise in medical oncology, surgical oncology, radiation oncology and radiology. It is crucial for highly specialized centers to collaborate via networks that aim to maintain uniformity in every aspect of treatment and rigorously gather patients’ data, from the first clinical evaluation to the last follow-up visit.

The Advanced International Mini-Invasive Surgery (AIMS) academy clinical research network aims to create a rectal cancer registry. This will prospectively collect the data of patients operated on for non-metastatic rectal cancer in high volume colorectal surgical units through a well design pre-fashioned database for non-metastatic rectal cancer, in order to take all multidisciplinary aspects into consideration.

**Methods/Design:** The protocol describes a multicenter prospective observational cohort study, investigating demographics, frailty, cancer-related features, surgical and radiological parameters, and oncological outcomes among patients with non-metastatic rectal cancer who are candidates for surgery with curative intent. Patients enrolled in the present registry will be followed up for 5 years after surgery.

**Discussion: **Standardization and centralization of data collection for neoplastic diseases is a virtuous process for patient care. The creation of a register will allow the control of the quality of treatments provided and permit prospective and retrospective studies to be carried out on complete and reliable high quality data. Establishing data collection in a prospective and systematic fashion is the only possibility to preserve the enormous resource that each patient represents.

## Introduction

There are nearly 125,000 new cases of rectal cancer diagnosed every year in Europe, representing one of the leading causes of cancer-related morbidity and mortality world-wide ^1^. Five decades ago, the prognosis of rectal cancer was poor, with locoregional cancer recurrence rates of up to 40% and 5-year survival rates of <50% for locally-advanced tumors ^2^. These disappointing outcomes were improved by innovations in surgical technique, multimodality therapy and education ^3^.

Total mesorectal excision (TME) remains the cornerstone in the treatment of non-metastatic rectal cancer. To achieve high quality TME, which is the key-factor for proper oncological resection, surgeons must respect well-known embryological planes, which were made famous as the boundaries of Heald’s Holy plane ^4^.

While the surgical principles for rectal cancer have changed little during the last decade, the novelties in the radiological study of the disease, as well as in the administration of neoadjuvant and adjuvant therapy, have largely modified the treatment of this neoplasm ^5^. Nowadays, the management of rectal cancer is multimodal and involves a multidisciplinary team of cancer specialists with expertise in medical oncology, surgical oncology, radiation oncology and radiology. Therefore, it is becoming crucial for highly specialized centers to design pre-fashioned databases for non-metastatic rectal cancer in order to take all multidisciplinary aspects into consideration. Previous attempts to establish rectal cancer registries, such as The Norwegian Rectal Cancer Project ^6^ and The Spanish Rectal Cancer Project ^7^, were based on the need to first extend adequate oncological treatment, and second to increase the use of minimally invasive surgery.

The Advanced International Mini-Invasive Surgery (AIMS) academy clinical research network aims to create a rectal cancer registry that will prospectively collect data of patients operated on for non-metastatic rectal cancer in high volume colorectal surgical units, maintaining uniformity in every aspect of the treatment and rigorously gathering patients data, from the first clinical evaluation to the last follow-up visit.

### Purpose

The aim of the AIMS academy clinical research network rectal cancer registry is to prospectively collect data from different minimally-invasive colorectal units in Northern Italy, with standardization of the pre-operative, intra-operative and post-operative management for patients operated on for non-metastatic rectal cancer with curative intent.

The primary outcome is to prospectively collect short and long term oncological outcomes. The second outcome is to collect information on the compliance of patients to oncological treatments, both in neoadjuvant and adjuvant settings and their quality of life.

## Protocol

### Study design

This protocol describes a multicenter prospective observational cohort study, investigating demographics, cancer-related features and oncological outcomes among patients who are non-metastatic rectal cancer candidates for surgery with curative intent. Patients enrolled in the present registry will be followed up for 5 years after surgery. All participating centres are public tertiary non-academic hospitals of northern Italy.

The study was approved by the Comitato Etico Scientifico Milano Area 3 (protocol number 295-052019). The study protocol has been registered as an observational study at ClinicalTrials.gov: NCT04045236 (first received, 3 August 2019). All participating centres received approval from local ethics committees.

### Patients and eligibility criteria

Patients receiving the diagnosis of non-metastatic rectal cancer and the indication for a curative treatment will be enrolled in the registry. The target population will consist of all patients enrolled in the participating centers from the start of the rectal cancer registry on. Patients will be identified through their medical record numbers. One investigator in each center will obtain written informed consent from each patient and keep the patients updated on data collection.

Inclusion criteria: 1) histologically proved adenocarcinoma of the rectum; 2) patient aged > 18 years old; 3) indication for surgical resection with curative intent.

Exclusion criteria: need for emergency surgery, palliative operation or metastatic disease at presentation.

### Data collection

Demographic information and anamnesis with a focus on oncologic history will be recorded at the first outpatient visit, together with a complete clinical examination. Data regarding the symptoms of presentation will be collected and categorized as haemorrhagic framework, alteration of bowel habits or pain. Every patient will undergo a pre-operative staging (see Extended data: CRF1) with chest-abdominal computerized tomography scan with intravenous contrast, complete colonoscopy, pelvic magnetic resonance (MR) imaging and endorectal ultra sound (EUS) examination. Blood sample with serum level of CEA, CA 19.9 and a full nutritional panel will be collected and analysed. Charlson Comorbidities Index adjusted for age ^8^ will be calculated for every patient, while those >70 years old will be assessed for frailty risk using the modified Frailty Index (mFI) described by Robinson *et al.* ^9^ (see Extended data: CRF1).

All data regarding radiation doses received, total amount of chemotherapy administered and number of cycles, toxicities or adverse reaction and possible reasons for not completed treatment schedule will be collected for all the patients with locally advance rectal cancer, who received neo adjuvant chemo-radiotherapy. Radiological restaging after neoadjuvant treatment comprises MR imaging, EUS and colonoscopy. Radiological response to neoadjuvant treatment will be measured following a uniform score system among all centers involved ^10^. Endoscopic assessment of tumour regression will be also recorded ^11^ (see *Extended data:* CRF2).

Intraoperative analysed parameters will be included in the registry (see Extended data: CRF3), with special attention to technical aspects of surgical procedures, such as level of inferior mesenteric artery ligation, type of energy device used, number and type of cartridge, and size of circular stapler and all other variables detailed in the Clinical Trials registration.

Histopathological examination will be performed according to WHO 2010 guidelines ^12^. Macroscopic evaluation of the resected specimen will be classified according to the Quirke score ^13^, while pathologic regression grade will be estimated according to a five-point scoring system ^14^. Mismatch repair status will be reported when analysed (see *Extended data:* CRF3).

Post-operative complications will be reported according to the Clavien-Dindo scale ^15^. Length of stay and eventual post-discharge complications will be evaluated and recorded. Application of an ERAS protocol will be considered only for at least 80% of ERAS colorectal items satisfied (see *Extended data:* CRF4) ^16^.

Indication to adjuvant treatment will be defined within a multidisciplinary setting. Regarding adjuvant chemotherapy, all data of interest such as number of cycles, toxicities and possible early interruption of the treatment will be collected as previously shown in the neoadjuvant setting. Oncological follow-up will be performed according to National Comprehensive Cancer Network guidelines ^17^. One investigator in each center will carry out the follow up. Functional follow up will be done yearly according to the Low Anterior Resection Syndrome Score (see *Extended data:* CRF5) ^18^.

### Data management

Data will be collected daily using a pre-fashioned REDCAP database by one physician for each hospital and referred to a research fellow (GMM) who will monitor the included data for all institutions. Pre-fashioned CRFs are available as *Extended data*. There will be regular contact between the study coordinators and the participating centers through scheduled meetings every three months. A data manager (GP) will regularly control the quality of the data provided.

### Dissemination of the registry

All researchers will be able to access the data uploaded. Data will be hosted by the AIMS Academy. All researchers will be able to use collected data to write scientific articles or to plan surgical audits.

### Study status

The registry has been enrolling patients since January 2019.

## Discussion

The primary aim of this registry is to prospectively collect data from different minimally invasive colorectal units in Northern Italy with a standardization of the pre-operative, intra-operative and post-operative management for patients operated on for non-metastatic rectal cancer with curative intent.

Standardization and centralization of data collection for neoplastic diseases is a virtuous process for patient care. The creation of a register allows the control of the quality of treatments provided and permits prospective and retrospective studies to be carried out on complete and reliable high quality data.

In the last few years, the need to raise the quality of care for rectal cancer patients has been reported in numerous studies ^19^, as well as the clear association between hospital volumes and outcomes after rectal surgery ^20^. Speaking a common language in such a complex field is no longer a benefit but rather the only way to face new challenges waiting for us in the near future.

Due to the aging population, the number of frail patients affected by rectal cancer is expected to grow ^21^. As a consequence, the identification of frail patients and the need to search for tailored management able to prevent adverse complications and to improve clinical outcomes of this population will play a fundamental role in oncological surgery in the years to come ^22^. Thus, all methods available to screen a patient for frailty, such as the Mini Cog test, the Katz Index of Independence in Activities of Daily Living (ADL), and the Timed Up and Go (TUG) test must become an integrated part of daily clinical work ^23, 24^.

The accuracy achieved by pre-operative MR imaging during the last decade ^25^ has lead to important results in both preoperative staging and radiologic response evaluation after neo-adjuvant therapy ^26^. Indeed, radiologic restaging is increasingly involved in the therapeutic decision-making process ^27^. Thus, structuring a synoptic and uniform MR report must become a prerogative in management of rectal cancer patients.

As mentioned in the study protocol section, intra-operative data concerning which type of devices or staplers used during surgery will be recorded in the registry, allowing us to look for possible correlations with clinical outcomes. A well-structured prospective analysis among high volume units will help to define the real complication rate after rectal cancer surgery, which has been usually derived retrospectively and therefore potentially underestimated ^28^.

Regarding surgical expertise among the centers involved, continuous monitoring of the integrity of the resected specimens should definitely increase the overall quality of surgery.

Detailed analysis of the compliance to adjuvant chemotherapy for locally advanced rectal cancer is extremely important considering recent data reported in the literature ^29, 30^. The unexpected low level of compliance reported in these previous case series has questioned the traditional administration of adjuvant chemotherapy, searching for new strategies for locally advanced rectal tumours, such as total neoadjuvant chemotherapy ^3^.

## Conclusions

The creation of a registry for patients operated on for non-metastatic rectal cancer is a necessary requirement. Establishing data collection in a prospective and systematic fashion is the only possibility to preserve the enormous resource that each patient represents.

## Data availability

### Underlying data

No underlying data is associated with this article.

### Extended data

Zenodo: Rectal cancer AIMS Academy clinical research network registry, http://doi.org/10.5281/zenodo.3463627 ^31^

This project contains the following extended data:

-    CRF1: Patient’s information and cancer staging form.

-    CRF2: Neo-adjuvant chemoradiotheraphy and cancer restaging form.

-    CRF3: Surgery, surgical outcomes and pathological examination form.

-    CRF4: Adjuvant chemotherapy form.

-    CRF5: Oncological follow-up form.

Data are available under the terms of the Creative Commons Zero "No rights reserved" data waiver (CC0 1.0 Public domain dedication).
