# Supplementary material for: Creation of a rectal cancer registry in Italy by the Advanced International Mini-Invasive Surgery (AIMS) academy clinical research network
Source: F1000Res. 2019 Oct 10;8:1736. [Version 1] doi: 10.12688/f1000research.20702.1 (PMC6833985; doi:10.12688/f1000research.20702.1)
Supplement: Peer review history

# Peer review: Reviewer response for version 1

**DOI:** 10.5256/f1000research.22769.r55725

**Published:** 2019-11-14

**Authors:** Fabian Grass

**Affiliations:** 1 Department of Colon and Rectal Surgery, Mayo Clinic, Rochester, MN, USA; 2 Lausanne University Hospital CHUV, Lausanne, Switzerland

The Northern Italian research group presents a study protocol detailing specifics of a rectal cancer registry, which consists of a multicentric collaboration aiming to gather reliable, prospectively collected data for rectal cancer research. The authors need to be congratulated for their initiative. Multi-institutional collaborations are needed in the rapidly evolving field of multi-modal treatment of locally advanced rectal cancer. The authors emphasise new challenges of an ageing, frail population. Radiotherapy- or surgery-sparing strategies may represent interesting alternatives to conventional treatment schemes.

 Overall the protocol is clear and concise. I would like to suggest expanding on the following points:

- Multi-institutional collaborations are challenging, since dealing with a heterogeous patient- and provider population, surgical and perioperative care. How do the authors account for that?
- Standardization: Do all centers use similar treatment protocols? How standardized is the surgical approach? Do all centers perform robotic proctoectomy? How experienced are participating surgeons? Are all participating centers teaching facilities?
- I think the authors may expand on complication assessment. Who is assessing complications, are these institution-specific abstractors or do centers dispose of surveillance tools, i.e. for surgical site infections?
- How do the authors ascertain data quality? National data registries ideally need (independent) audit and validation. Who is auditing data accuracy?

I confirm that I have read this submission and believe that I have an appropriate level of expertise to confirm that it is of an acceptable scientific standard.

# Peer review: Reviewer response for version 1

**DOI:** 10.5256/f1000research.22769.r55011

**Published:** 2019-11-05

**Authors:** Abe Fingerhut

**Affiliations:** 1 Section for Surgical Research, Department of Surgery, Medical University of Graz, Graz, Austria

This registry is unique and essential to reseach in Italy.

 The Advanced International Mini-Invasive Surgery (AIMS) academy clinical research network has a major role in this multicenter prospective observational cohort study, I agree that standardization and centralization of data collection for neoplastic diseases is essential for patient care. The creation of a register will allow the control of the quality of treatments provided and permit prospective and retrospective studies to be carried out on complete and reliable high quality data.

I confirm that I have read this submission and believe that I have an appropriate level of expertise to confirm that it is of an acceptable scientific standard.

# Peer review: Reviewer response for version 1

**DOI:** 10.5256/f1000research.22769.r55012

**Published:** 2019-10-17

**Authors:** Giovanni Cesana

**Affiliations:** 1 Department of Surgery, S.I.C.OB. (Italian Society of Bariatric Surgery) Referral Center for Bariatric Surgery, San Donato Group, Policlinico San Marco, Zingonia, Italy; 2 Policlinico San Marco, Zingonia, Italy

This paper describes the creation of a national registry for non metastatic rectal cancer. The main issue is a high quality standardization of rectal cancer multimodal treatment, staging and follow up.

 I think that the core of the project is well explained and clarified. Additional materials are clear and quite user friendly.

 The methods are well described and expanded enough.

 The need for well designed databases on rectal cancer is clearly expressed by the literature.

 I believe that this registry represents a high quality attempt in the direction of a centralized and appropriate treatment for rectal cancer.

I confirm that I have read this submission and believe that I have an appropriate level of expertise to confirm that it is of an acceptable scientific standard.
